# Genetically Encoded Microtubule Binders for Single-Cell Interrogation of Cytoskeleton Dynamics and Protein Activity

**DOI:** 10.1021/acssensors.4c01167

**Published:** 2024-08-15

**Authors:** Joseph Zhou, Xiaoxuan Liu, Dekai Zhang, Guolin Ma

**Affiliations:** † Institute of Biosciences and Technology, Texas A&M University, Houston, Texas 77030, United States; ‡ ORBIT Platform, The University of Texas MD Anderson Cancer Center, Houston, Texas 77054, United States

**Keywords:** microtubule, cytoskeleton, biosensor, protein−protein interactions, drug discovery, high-throughput screening

## Abstract

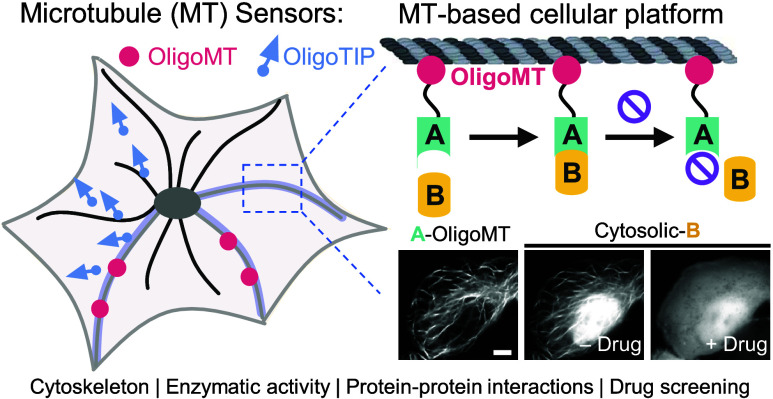

Microtubule (MT) dynamics is tightly regulated by microtubule-associated proteins (MAPs) and various post-translational modifications (PTMs) of tubulin. Here, we introduce OligoMT and OligoTIP as genetically encoded oligomeric MT binders designed for real-time visualization and manipulation of MT behaviors within living cells. OligoMT acts as a reliable marker to label the MT cytoskeleton, while OligoTIP allows for live monitoring of the growing MT plus-ends. These engineered MT binders have been successfully utilized to label the MT network, monitor cell division, track MT plus-ends, and assess the effect of tubulin acetylation on the MT stability at the single-cell level. Moreover, OligoMT and OligoTIP can be repurposed as biosensors for quantitative assessment of drug actions and for reporting enzymatic activity. Overall, these engineered MT binders hold promise for advancing the mechanistic dissection of MT biology and have translational applications in cell-based high-throughput drug discovery efforts.

Microtubules (MTs), as key dynamic structural elements of the cytoskeleton, play a pivotal role in multiple cellular functions that are essential for maintaining cell shape, facilitating cell reproduction and division, enabling cell signaling, supporting intracellular transport, and promoting cell movement.^1−3^ The MT cytoskeleton undergoes dynamic changes in a spatially and temporally controlled manner due to the coordinated actions of protein regulators including microtubule-associated proteins (MAPs) and molecular motors.^2−6^ Notably, microtubule plus end-tracking proteins (+TIPs) contain a highly conserved S/T-x–I-P (SxIP) motif that specifically binds to the end-binding (EB) proteins and can thus be used to track microtubule plus ends.^3,4,7^ Tubulin post-translational modifications (PTMs) enable microtubules displaying diversified functions within cells and organisms.^8,9^

Deregulation of MT dynamics and PTMs may lead to genome instability and cell cycle arrest and is often associated with human diseases, such as cancer,^10^ cardiovascular disease,^11^ and neurological disorders.^12^ Visualizing the temporal and spatial distribution, PTMs and dynamics of MTs are essential to understand the function of MTs in various cell types during cell growth and differentiation.^1−3^ Currently, the existing tools for monitoring the MT cytoskeleton and its dynamics include the ectopic expression of fluorescent tubulin proteins,^13^ antibody-based immunostaining with fixed cells, and fluorophore-conjugated chemical reagents that covalently label tubulin.^14^ Nonetheless, overexpression or chemical modifications of tubulin tend to perturb the host MT polymerization,^10,13^ whereas immunostaining requires the use of fixed cells rather than living cells. More robust and convenient methods that allow for real-time detection of microtubule dynamics while minimizing disturbance to the host cells are required to expedite research on the cytoskeleton.

Microtubule-associated proteins commonly exhibit structurally conserved features, encompassing MT association/binding domains (MADs), coil–coil domains (CC), and functional/enzyme domains.^2,5^ Dimerization or oligomerization mediated by CC domains is crucial for MAPs to bind to microtubules, enabling them to execute their functions.^2,5^ Previously, we systematically analyzed and screened a range of MT-associated binding domains or motifs, leading to the development of two genetically encoded tags, designated as “MoTags”, to enable in-cell probing of their oligomeric states.^15^ Herein, we employ synthetic biology approaches and integrate the well-characterized oligomerization domain (OD) of p73 with MT association/binding domains. Our engineered MT tracers allow us to track and manipulate the MT cytoskeleton and MT plus ends with simple methods under physiological conditions in living cells. This system could aid the mechanistic dissection of how tubulin PTMs impact MT stability, morphology, and dynamics.

Protein–protein interactions (PPIs) are vital components of cellular signal transduction networks.^16^ Developing methods to detect PPIs within living cells is essential for understanding physiological and pathological processes and facilitating therapeutic drug screening.^17,18^ While conventional fluorescence or bioluminescence resonance energy transfer (FRET or BRET) assays are widely used for intracellular PPI detection, they rely on proteins maintaining appropriate orientations and require complex equipment.^17,18^ In contrast, our OligoMT platform, leveraging the widespread distribution of microtubules within cells, enables real-time and direct visualization of intracellular PPIs without such constraints. This platform effectively illustrates the interaction between p53 and MDM2 and assesses the inhibitory effects of small molecules and peptides on these interactions. Thus, the OligoMT-based PPI platform shows significant promise for the high-throughput screening of PPI inhibitors within live cells.

## Results

### Design of OligoMT for Real-Time Monitoring of the MT Cytoskeleton

**Figure 1:**
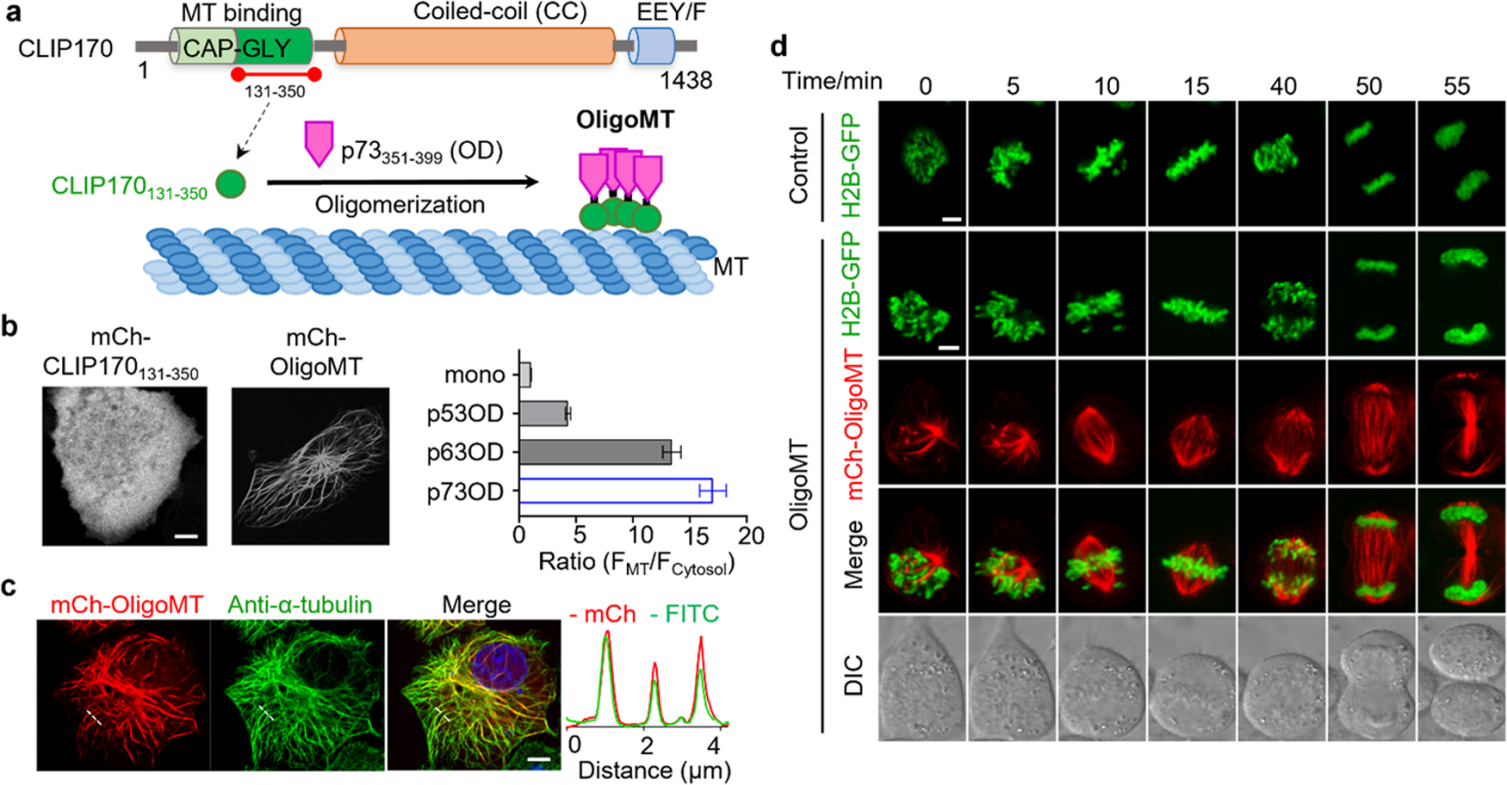
OligoMT designed for real-time visualization of the microtubule cytoskeleton. (a) Schematic illustrating the fusion of the N-terminal MT-binding region (aa 131–350) of CLIP170 with the p73 oligomerization domain of (OD; aa 351–399) to enable MT binding. CAP-GLY, cytoskeleton-associated protein glycine-rich; MT, microtubule; and OD, oligomerization domain. (b) Confocal images showing the distribution of the mCherry (mCh)-tagged MT-binding region of CLIP170 (aa 131–350) and OligoMT (p73_351–399_-CLIP170_131–350_ chimera) in HeLa cells. The bar graph on the right shows the *F*_MT_/*F*_cytosol_ ratios in HeLa cells expressing oligomerization domains from the p53 family fused to CLIP170_131–350_ (*n* = 30 cells; mean ± SEM). (c) Confocal images of HeLa cells transfected with mCh-OligoMT (red) and costained with an anti-α-tubulin antibody (green) and DAPI (blue). The red and green fluorescence intensities across the dashed line were plotted alongside the images to illustrate the extent of signal overlap. (d) Time-lapse confocal images depict cell mitosis in HeLa cells expressing H2B-GFP (green) and mCh-OligoMT (red). The bottom panel displays the differential interference contrast (DIC) views of the assayed cells. HeLa cells expressing mCh-OligoMT exhibited mitotic progression comparable to the control group (also, see Supporting Video S2), indicative of its minimal perturbation to host cells. Scale bar, 5 μm.

CLIP170, a microtubule “plus end-tracking protein”, plays crucial roles in regulating microtubule dynamics and facilitating dynactin localization and transportation of subcellular organelles.^19,20^ Structurally, it comprises two N-terminal microtubule-binding CAP-GLY domains, a central coiled-coil (CC) region, and C-terminal metal-binding motifs. The coiled-coil region (∼1000 residues) facilitates CLIP170 dimerization, thereby enhancing its targeting toward microtubules.^19,20^ We hypothesized that replacing the CLIP170 coiled-coil region with an oligomerization module would enhance MT binding when fused to the CAP-GLY domain (Figure 1a). We previously mapped the microtubule binding of CLIP170 and identified a highly effective MT-binding region, CLIP170_131–350_.^15^ Herein, we utilized fluorescence live-cell imaging to screen a range of constructs that fuse the oligomerization domains (OD)^21^ of the p53 family to the microtubule-binding domain (MTBD) CLIP170_131–350_ in HeLa cells (Figures 1b and S1). Unlike the monomeric version of CLIP170_131–350_, which displays a uniform distribution throughout the cytosol, oligomeric CLIP170_131–350_ effectively labels microtubules, providing clear visualization of MT cytoskeletal filaments. Next, we quantitatively compared the oligomerization domains (OD) of p53, p63, and p73 by analyzing the ratio of fluorescence intensity between labeled microtubules and the cytosol (*F*_MT_/*F*_cytosol_; Figure 1b). Compared to p53_OD_ and p63_OD_, the fusion construct of p73_OD_ with CLIP170_131–350_ demonstrated exceptional microtubule distribution, with the *F*_MT_/*F*_cytosol_ value ranging between 4 and 5. Therefore, we chose the chimera made of p73_OD_-CLIP170_131–350_ (termed OligoMT) for further characterization.

To validate the accurate labeling of the MT cytoskeleton, we conducted immunostaining analysis on HeLa cells expressing mCherry-OligoMT using an anti-α-tubulin antibody. We observed robust colocalization of mCherry-OligoMT with microtubules in fixed HeLa cells (Figure 1c), supported by quantitative analysis of the red and green fluorescence intensities along the dashed line plotted adjacent to the images. These findings suggest that genetically encoded, single-component OligoMT can be applied for real-time labeling of the MT cytoskeleton (Supporting Video S1). We quantified the expression levels and post-transfection times of OligoMT to determine the optimal conditions for subsequent experiments. Based on our results (Figure S2), we selected a medium expression level (with 100 ng of plasmid) and acquired the images at 24 h after transfection. To demonstrate the versatility of our OligoMT, we expressed it in four additional cell lines derived from various tissues or organs (Figure S3), including HEK-293 (human embryonic kidney cells), U-2 OS (human osteosarcoma cells), HCT116 (human colorectal carcinoma cells), and COS-7 (CV-1 African green monkey kidney fibroblast cells). All four cell lines exhibited excellent microtubule labeling (Figure S3).

### OligoMT as a Reliable MT Marker without Disturbing MT Dynamics

Microtubules exhibit dynamic polymerization and depolymerization, a critical property that is essential for cellular functions such as mitotic spindle organization and chromosome segregation.^3,6^ To further demonstrate OligoMT as a robust live-cell MT marker, we monitored cell mitosis from prophase to telophase in HeLa cells expressing GFP-tagged core histone 2B (H2B-GFP) with or without the coexpression of mCh-OligoMT using time-lapse confocal microscopy (Figure 1d and Supporting Video S2). We simultaneously evaluated the potential impact of OligoMT overexpression on microtubule dynamics and the mitotic chromatin phase transition. We found that cells overexpressing OligoMT displayed a mitotic process akin to those expressing solely H2B-GFP, indicating that the OligoMT expression did not affect the dynamics of mitotic progression. Meanwhile, OligoMT proves to be an excellent marker for visualizing cell mitosis through the reliable labeling of microtubules. Furthermore, we assessed the viability and cell cycle progression of cells expressing OligoMT. We found that approximately 95% of the cells remained viable, and overexpression of OligoMT did not seem to exhibit appreciable perturbation to cell cycle (Figure S4).

EB1, a microtubule-binding protein, localizes to the growing plus end of microtubules in cells, serving as a marker for their dynamics.^22^ To investigate whether OligoMT altered microtubule plus-end dynamics, we tracked the movement trajectories of EB1-GFP comets over time in HeLa cells with or without the coexpression of mCh-OligoMT (Figure S5 and Supporting Video S3). We did not detect notable difference in EB1 movement velocity between the control group (EB1 alone) and the OligoMT group (with EB1 coexpression). Collectively, these findings suggest that the overexpression of OligoMT does not appear to interfere with microtubule dynamics or affect cell mitosis or viability.

### OligoMT for Real-Time Monitoring of MT Depolymerization

**Figure 2:**
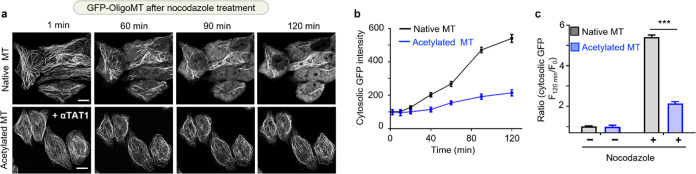
OligoMT used to monitor nocodazole-induced damages to native or acetylated microtubule in HeLa cells. (a) Time lapse images showing HeLa cells expressing GFP-OliogMT without (top) or with αTAT1 coexpression for tubulin acetylation (bottom). Both groups were subjected to treatment with 2 μM nocodazole. The dispersal of GFP-OligoMT throughout the cytosol indicates nocodazole-induced MT depolymerization. Scale bar, 10 μm. (b) Measurements of dispersed cytosolic GFP intensities (indicator of damaged MT) following nocodazole treatment in cells shown in (a). (c) Quantification of dispersed cytosolic GFP intensities before and after 2 μM nocodazole treatment for 2 h. *n* = 35 cells from three independent experiments (mean ± SEM; ***P < 0.001; paired Student’s *t* test).

To evaluate the ability of OligoMT monitoring microtubule kinetics in live cells, we employed OligoMT to track the depolymerization of microtubules triggered by microtubule-depolymerizing drugs like nocodazole.^23^ Acetylated microtubules are frequently regarded as exhibiting greater stability and longevity in comparison to unmodified counterparts, as they demonstrate resistance to mild nocodazole treatment.^24^ To validate the functional consequences of microtubule acetylation, we selected α-tubulin acetyltransferase (αTAT1),^24^ a key enzyme responsible for acetylating α-tubulin at Lys40, to induce microtubule acetylation. HeLa cells expressing GFP-tagged OligoMT, with and without the coexpression of αTAT1, were subjected to individual treatment with 2 μM nocodazole (Figure 2). Following the addition of nocodazole, we observed microtubules initiating shrinkage from their periphery, with gradual disassembly occurring within 120 min (Figure 2a). By measuring the dispersed cytosolic GFP intensity, which serves as an indicator of damaged microtubules, we monitored the process of microtubule depolymerization induced by nocodazole (Figure 2b). HeLa cells expressing αTAT1 exhibited a pronounced resistance to the nocodazole-induced destabilization of the microtubule cytoskeleton during 120 min treatment. In HeLa cells with native microtubules, the cytosolic GFP intensity increased 6-fold following nocodazole treatment. By comparison, in αTAT1-expressing cells bearing more acetylated microtubules, the microtubule network remained largely intact, with dispersed cytosolic GFP intensity increased by only 2-fold (Figure 2c). Overall, our study demonstrates the utility of OligoMT for real-time monitoring of microtubule dynamics and illustrates a protective role of αTAT1 in safeguarding the stability of the microtubule cytoskeleton.

### OligoTIP as a Genetically Encoded MT Plus-End Tracker

**Figure 3:**
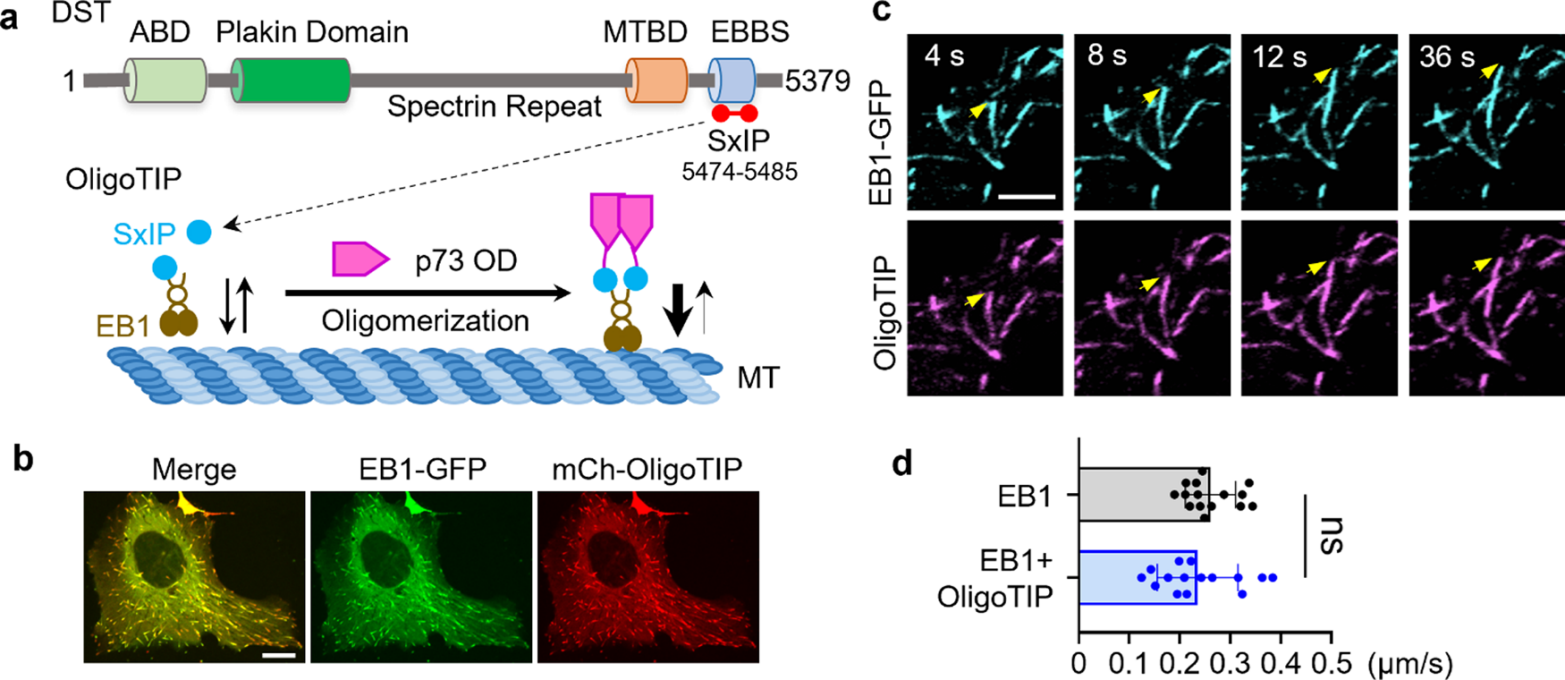
OligoTIP was designed for real-time tracking of microtubule plus-ends. (a) Schematic showing the design and binding of OligoTIP to EB1 to track MT plus-ends. OligoTIP was constructed by fusing p73-OD with the EB1-binding SxIP motifs (aa 5474–5485) derived from dystonin (DST). ABD, actin-binding domain; MTBD, microtubule-binding domain; and EBBS, EB1/EB3-binding site containing a Ser-X-Ile-Pro motif. (b) Confocal images of HeLa cells expressing mCh-OligoTIP (red) and EB-GFP (gren). Scale bar, 5 μm. (c) Enlarged time-lapse images showing the tight colocalization of mCh-OligoTIP (magenta) with EB1-GFP (cyan). The arrows indicate the growing tips of microtubule. Also, refer to Supporting Video S5. Scale bar: 1 μm. (d) Quantification of comet velocity of EB1 with and without OligoTIP coexpression in HeLa cells. *n* = 15 cells from three independent experiments (mean ± SEM; ns, not significant; paired Student’s *t* test).

EB1 serves as a marker for MT plus-ends in certain scenarios.^22^ However, overexpression of EB1 can impact microtubule dynamics by increasing growth rates, stabilizing microtubules against catastrophes, or perturbing the localization of other plus end-tracking proteins.^25^ It is crucial to design a genetically encoded MT plus-end tracker that minimally interferes with the intrinsic dynamics of MT plus-ends. We previously dissected the microtubule +TIP localization signals in various oligomeric states, which included the S/T-x–I-P (SxIP) consensus motif found in three microtubule plus end-tracking proteins (+TIPs).^4,26,27^ These +TIPs utilize the SxIP motif to track the ends of extending microtubules through direct interactions with EB1, whose C-terminal EB-homology (EBH) domain recruits +TIPs.^28^ Among these, the dystonin SxIP-containing peptide (DST_5474–5485_) exhibited the most remarkable pulse-end tracking capability. Consequently, we fused this 12-mer peptide with the p73 oligomerization domain (OD) and generated the +TIP tracking sensor, OligoTIP (Figure 3a), which tracks the tips of the microtubule network labeled by GFP-OligoMT (Supporting Video S4).

When coexpressed in the same cell, mCh-OligoTIP colocalized with EB1-GFP (Figure 3b,c and Supporting Video S5), with the fluorescence intensity profiles of both comets remaining largely consistent at growing microtubule ends (Figure 3c). These findings clearly indicate that OligoTIP tracks +TIPs through EB1. To assess whether OligoTIP influenced the behavior of growing microtubule ends, we compared the microtubule tip tracking velocities of free EB1-GFP comets and EB1-GFP comets in the presence of mCherry-OligoTIP (Supporting Video S5). No significant difference was observed between the velocities of EB1 comets with and without the coexpression of mCherry-OligoTIP (Figure 3d). Together, these results confirmed OligoTIP as an excellent sensor for EB1-marked +TIPs, which allows the real-time tracking of growing microtubule plus-ends in living cells.

### MT Tracers for Real-Time Assessment of Drug Action and Enzymatic Activity

**Figure 4:**
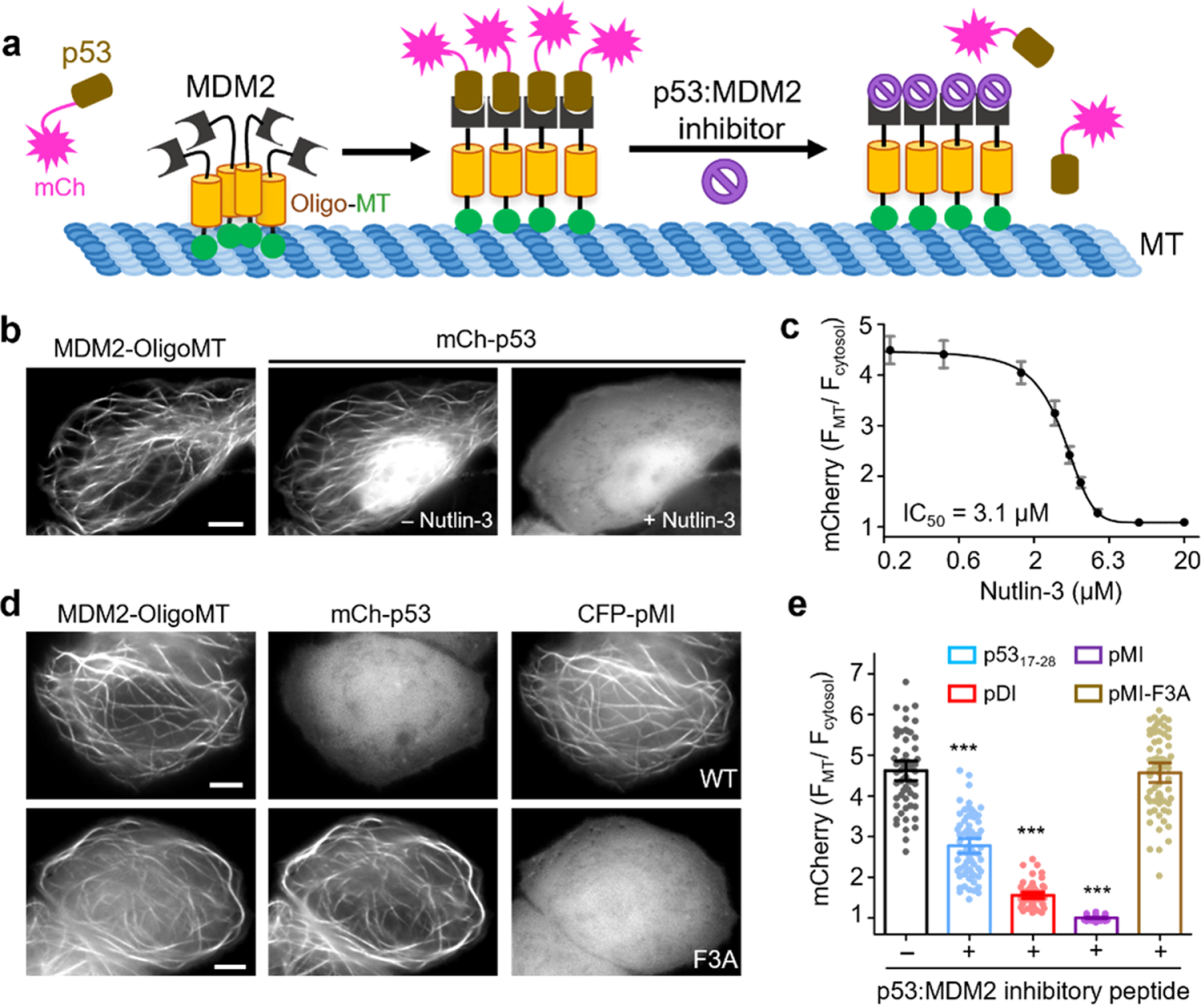
OligoMT for quantitative evaluation of inhibitors targeting the p53-MDM2 interaction. (a) Schematic illustrating the design of an OligoMT-based assay to assess the cellular efficacy of p53-MDM2 inhibitors. MDM2-OligoMT oligomerizes to label MTs, accompanied by subsequent p53 recruitment to MT via the MDM2:P53 interaction. Upon the addition of MDM2 inhibitors, p53 is released from MDM2-bound microtubules, resulting in a more diffuse distribution of p53 throughout the cytosol. (b) Confocal images of HeLa cells coexpressing MDM2-GFP-OligoMT (left) and mCh-p53 before (middle) or after (right) treatment with 5 μM nutlin-3. Also, refer to Supporting Video S6. (c) MT–over–cytosol ratio of mCherry signals plotted as a function of nutlin-3 concentrations (IC_50_: 3.1 ± 0.2 μM). *n* = 50 cells from three independent experiments (mean ± SEM). (d) Confocal images of HeLa cells coexpressing MDM2-GFP-OligoMT (left), mCh-p53 (middle), and CFP-tagged p53-MDM2 inhibitor peptides (right panels; top, WT pMI; bottom, pMI mutant F3A lacking the suppressive activity). (e) MT–over–cytosol ratio of mCherry signals to gauge the suppressive effects of peptide inhibitors on p53:MDM2 association. Each column represents 5–6 measurements per cell. At least 59 cells from three independent experiments were analyzed (mean ± SEM; ***P < 0.001; paired Student’s *t* test). Scale bars, 5 μm.

Next, we explored the potential of utilizing OligoMT or OligoTIP as a platform to investigate protein–protein interactions (PPIs) and drug screening (Figure 4a). We used the p53-MDM2 interaction as a test case given the established 1:1 stoichiometry and the availability of well-characterized inhibitors (Figure 4a), including both small molecules (such as nutlin-3) and polypeptides (e.g., a 12-mer peptide derived from p53_17–28_, pMI, or pDI).^29^ In HeLa cells coexpressing mCherry-tagged p53 (mCh-p53) and MDM2-GFP-OligoMT, we observed the colocalization of these two proteins along the microtubule cytoskeleton (Figures 4b and S6a). As the MDM2 inhibitor nutlin-3 was introduced into the cells, mCh-p53 rapidly dissociated from its bound state on microtubules to the cytoplasm. Nutlin-3 displaced the binding of p53 to MDM2 (IC_50_: ∼3 μM; Figures 4b,c, S6b,c, and Supporting Video S6). A comparable translocation from microtubules to the cytosol was observed in HeLa cells with the coexpression of potent p53-MDM2 inhibitor peptides such as pMI (Figures 4d,e and S6b). As a rigorous control, a mutant peptide (pMI-F3A) with diminished MDM2 inhibitory activity demonstrated an inability to displace mCh-p53 from microtubules (Figures 4e and S6a). Clearly, the tethering of a bait protein to the microtubule cytoskeleton presents a distinct subcellular readout that can be leveraged for the quantitative assessment of its association with potential binding partners. This platform holds promise for further optimization aimed at screening compounds capable of disrupting protein–protein interactions.

**Figure 5:**
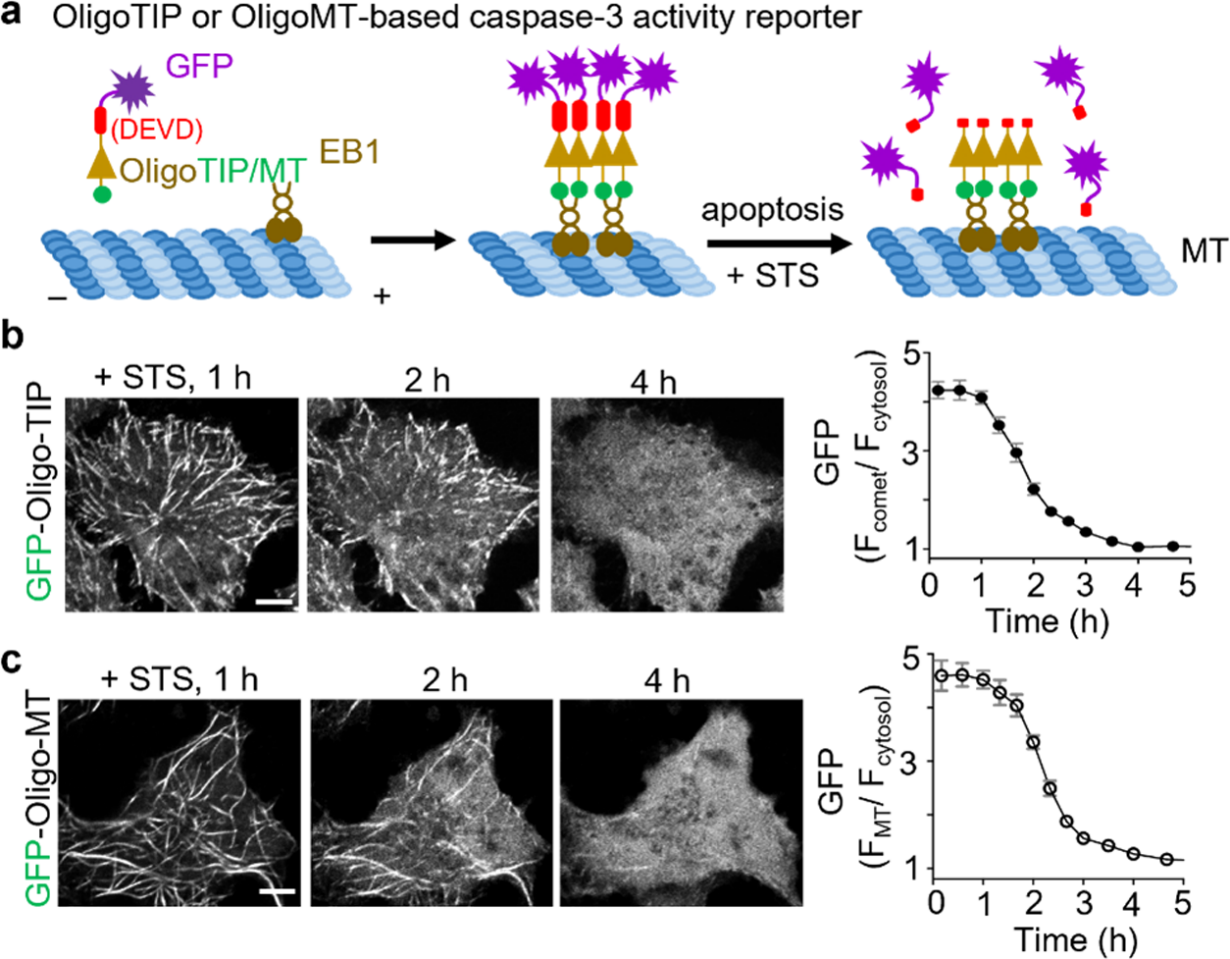
Real-time monitoring of caspase-3 activity in living cells utilizing OligoTIP or OligoMT. (a) Schematic depicting the design of assays based on OligoTIP or OligoMT for monitoring caspase-3 activity in HeLa cells. GFP-DEVD-OligoTIP or GFP-DEVD-OligoMT tracks MT plus ends or labels the MT cytoskeleton, respectively. Upon apoptotic stimulation with staurosporine (STS) to activate caspase-3 and subsequent cleavage at DEVD, the GFP tag is cleaved away, thus leading to its dispersion into the cytoplasm and subsequent loss of MT tracking. (b) Time lapse confocal images of a typical HeLa cell expressing the caspase-3 reporter, GFP-DEVD-OligoTIP, following the addition of 1 μM STS. The time course is shown on the right. *n* = 14 cells (mean ± SEM). Scale bars, 5 μm. (c) Time lapse confocal images of a single HeLa cell expressing GFP-DEVD-OligoMT after incubation with 1 μM STS. The time course of MT–over–cytosol GFP signals is shown on the right. *n* = 14 cells (mean ± SEM). Scale bars, 5 μm.

In parallel, we tested the idea of employing OligoTIP or OligoMT as a fusion tag for assessing caspase-3-mediated apoptotic activity in living cells (Figure 5a).^30^ To achieve this, we fused a classical caspase-3 cleavage site, DEVD, along with GFP and flanking linkers to the N-termini of OptoTIP (Figure 5b) or OptoMT (Figure 5c). The chimeric proteins GFP-DEVD-OligoTIP and GFP-DEVD-OligoMT tracked the microtubule plus ends and bound to the microtubule cytoskeleton, respectively. Upon the addition of staurosporine (STS) to activate caspase-3 and induce apoptosis, we observed the gradual loss of MT tip tracking or MT binding within 2–3 h (Figure 5b,c), accompanied by the simultaneous increase in dispersed cytosolic GFP signals (Figure S7). Hence, our engineered genetically encoded microtubule tracers could be repurposed to report enzymatic activity in real time within single cells.

## Discussion

Visualizing microtubules and dissecting their regulatory mechanisms hold significant importance in physiological and medical contexts.^1,3,6,11,12^ Enhanced live cell imaging techniques, coupled with widespread application of genetically encoded biosensors, offer unprecedented opportunities for elucidating the intricate architecture and functionality of the cytoskeleton within cells.^1,11^ In the present study, we have developed a suite of genetically encoded microtubule (MT) tracers, including OligoMT and OligoTIP. This toolkit enables real-time monitoring of the MT morphology and kinetics as well as tracking MT plus-ends in living cells, while circumventing the artifacts associated with conventional MT labeling techniques. Importantly, under our experimental conditions, the use of OligoMT and OligoTIP did not appear to adversely affect the cells, exerting a negligible perturbation to cell viability, mitosis, and divisions. Therefore, OligoMT or OligoTIP is well-suited for monitoring MT distribution, morphology, and dynamics in mammalian cells. Moreover, the mechanisms responsible for the formation and maintenance of microtubule networks in tissues yet remain to be fully understood, primarily due to the complexity of visualizing and probing three-dimensional microtubule arrays within tissues.^1,11^ OligoMT and OligoTIP offer additional tools for exploring microtubule networks in living cells.

In addition to its utility in examining the MT cytoskeleton network and kinetics, OligoMT holds potential applicability in studying protein–protein interactions and enzymatic function within living cells. Furthermore, these tools can be repurposed as high-throughput, live-cell drug-screening platforms. In mammals, tubulin represents about 3–4% of the total proteins in cells and up to 10% in the brain.^31^ Comprising α- and β-tubulin subunits, microtubules assemble into a rigid cylindrical structure with a diameter of ∼25 nm and a typical length spanning micrometers, forming an extensive network throughout the cell.^6^ Compared to subcellular organelle plasma membranes (PMs), which tend to favor reducing environments, and the mitochondria, which favor oxidative environments,^32^ microtubules serve as an ideal intracellular reaction site due to their pervasive distribution throughout the cytoplasm and lack of redox bias. Furthermore, the distinct structural morphology of microtubules facilitates easy differentiation within the cytoplasm, enabling straightforward identification of protein–protein interactions and quantitative analysis, thereby aiding in the establishment of high-throughput screening assays. This study showcases the utility of OligoMT for real-time monitoring of the MDM2-p53 interaction within living cells. Notably, both small molecules (such as nutlin-3) and peptides (such as pMI and pDI) effectively disrupt this interaction, liberating MT-bound p53 into the cytoplasm. This exemplifies the advantages of utilizing an OligoMT-based intracellular reaction platform. Additionally, our investigation extends the application of OligoMT and OligoTIP to evaluate the caspase activity. Such assays can be readily developed and customized to probe the functionality of other enzymes.

Screening for protein–protein interactions and drug–target actions on microtubules offers several advantages compared to other subcellular compartments, such as the plasma membrane (PM). First, PM is highly dynamic, constantly undergoing endocytosis, exocytosis, and lateral diffusion of proteins. These processes can complicate the study of stable interactions by creating fluctuating conditions and introducing variability. In contrast, microtubules, while also being dynamic, provide a more stable platform for studying PPIs due to their relatively predictable polymerization and depolymerization cycles. Second, the inner leaflet of the plasma membrane is highly negatively charged due to the presence of polyphosphoinositides. This negative charge can repel some negatively charged proteins, potentially leading to false negative results in PPI screening (such as the charge-complementary coiled coil interactions). On the other hand, the microtubule network, composed of tubulin proteins, does not exhibit such extreme electrostatic properties, reducing the likelihood of charge-based exclusion and thus providing a more inclusive environment for detecting PPIs. Third, the surface area available for binding to the microtubule network is large in a typical mammalian cell. This extensive surface area facilitates the attachment of multiple proteins and enhances the detection of PPIs. Additionally, the microtubule network spans throughout the cell, providing a widespread and accessible platform for observation of interactions in various cellular contexts.

## Methods

### Reagents and Antibodies

KOD Hot Start DNA polymerase was purchased from the EMD Millipore Corporation. Restriction endonucleases, NEBuilder HiFi DNA Assembly Master Mix, and T4 DNA ligase were obtained from New England BioLabs. Nocodazole (CAS, 31430-18-9), nutlin-3 (CAS, 548472-68-0), and staurosporine (CAS, 62996-74-1) were purchased from Sigma-Aldrich. These compounds were dissolved in DMSO and prepared as stock solutions (1–5 mg/mL). The anti-α-tubulin antibody was purchased from Santa Cruz Biotechnology (Cat No. SC-32293). Goat antimouse IgG highly cross-adsorbed secondary antibodies (conjugated with Alexa Fluor 488, Cat No. #A-11001) were purchased from Thermo Fisher Scientific.

### Plasmid Construction

pGFP-EB1 (Addgene no. 17234), CLIP170 (no. 54044), and MDM2-YFP (no. 53962) were purchased from Addgene. The oligomerization domains from the p53 family and EB1-binding SxIP motif (DST_5474–5485_) were directly synthesized as DNA oligos by Integrated DNA Technologies. To generate OligoMT and OligoTIP, the synthesized oligomerization domains and MT binding domains were cloned into modified pmCherry-C1 and pEGFP-C1 (Clontech). The N-terminal p53-binding domain (aa 1–119) of MDM2 was amplified and inserted into GFP-oligoMT. mCh-p53 was obtained by inserting the amplified p53 fragment into pmCherry-C1 between the *Hin*dIII and Xhol restriction sites. For CFP-tagged peptides, p53_17–28_, pDI, pMI, and pMI-F3A were directly synthesized by Integrated DNA Technologies and then inserted into pECFP-C1 at the *Hin*dIII and Xhol sites. To generate caspase reporters, the caspase-3 cleavage site DEVD with the flanking 8–10 aa linkers were introduced by using annealed primers and subsequently inserted into digested EGFP-OligoMT or EGFP-OligoTIP vectors at BsRGI and AgeI sites.

### Cell Culture and Transfection

HeLa cells were obtained from ATCC and cultured at 37 °C with 5% CO_2_ in complete cell culture medium. Cells were seeded in 35 mm glass-bottom cell culture dishes (MatTek). For transient transfection, 100–300 ng of plasmids was mixed with Lipofectamine 3000 in Opti-MEM medium (Invitrogen) by following the manufacturer’s instructions.

### Immunostaining

HeLa cells were grown on 35 mm glass-bottom dishes (MakTek). Upon reaching 60–80% confluency, the cells were transfected with mCh-OligoMT using Lipofectamine 3000. 18 h post-transfection, the cells were fixed with 4% paraformaldehyde (diluted from 16% stock) in PBS for 20 min and then permeabilized with 0.1% Triton X-100 in PBS for 10 min. After washing away the fixation solution with PBS and 0.1% Triton X-100 (PBST), we incubated the fixed cells with the blocking buffer (10% goat serum, Thermo Fisher, # 50062Z) for 1 h at room temperature, followed by the addition of a rabbit anti-α-tubulin antibody (1:200 dilution) overnight at 4 °C. After thorough washing with PBST, a secondary antibody (goat antimouse IgG Alexa Fluor 488; 1:500 dilution) was added to aid the visualization of tubulin. The nuclei were stained with DAPI. After extensive washing, the cells were kept in PBS and immediately imaged on a Nikon A1R confocal microscope at 60× magnification.

### Live-Cell Imaging and Image Analysis

Live-cell imaging was mainly performed on a Nikon Eclipse Ti-E microscope (Nikon Instruments) equipped with a Yokogawa W-1 dual spinning disk scan head or an A1R-A1 confocal module with LU-N4 laser sources (argon ion: 405 and 488 nm; diode: 561 nm) and CFI (chrome-free infinity) plan Apochromat VC series objective lenses (60× oil or 40× oil). In some experiments, the DeltaVision imaging workstation (GE Healthcare) equipped with a 100×/1.45 oil lens and a CoolSNAP EMCCD camera was used to obtain epifluorescence images. To monitor the labeling of the microtubule or tracking of microtubule plus ends in real time, HeLa cells were first transfected with mCh-OligoMT or mCh-OligoTIP and imaged 18 h post-transfection. To monitor the effect of nocodazole on microtubule dynamics, HeLa cells were plated on 35 mm glass-bottom cell culture dishes and transfected with GFP-OligoMT and with or without mCherry-tagged αTAT1. The dishes were subjected to incubation with 2 μM nocodazole. The confocal images were then acquired at various nocodazole treatment times (0–120 min) with excitations set at 488 and 562 nm.

To monitor the effects of MDM2 inhibitors on the p53-MDM2 interaction, HeLa cells were cotransfected with MDM2-GFP-OligoMT and mCherry-p53 and imaged by using a Nikon confocal microscope or a DeltaVision fluorescence microscope. Nutlin-3 was titrated into the medium (0–20 μM), while time-lapse imaging was carried out to monitor the drug-induced dissociation of mCh-p53 dissociating from microtubules. To monitor the behavior of MDM2 inhibitory peptides, CFP-tagged peptides (p53_17–28_, pMI, PDI, and pDI-F3A), MDM2-GFP-OligoMT, and mCherry-p53 were cotransfected into HeLa cells. The images were acquired on a DeltaVision fluorescence microscope with exposure times of 100 ms for YFP, 20 ms for CFP, and 50 ms for mCherry. To explore the use of OligoMT- or OligoTIP-based caspase-3 reporters, HeLa cells were transfected with GFP-(DEVD)-OligoMT or GFP-(DEVD)-OligoTIP and then imaged on a Nikon confocal microscope with a 40× oil lens. The time-lapse imaging was applied after addition of 1 μM staurosporine (STS) into the culture medium to elicit apoptosis. Multiple views were selected to monitor apoptosis for up to 6 h.

All of the acquired confocal images were analyzed by using NIS-Elements AR microscope imaging software (Nikon, NIS-element AR version 4.0). The cytosolic intensity of fluorescent protein was statistically analyzed by a semiautomatic image analysis tool of NIS-element AR software. The defined regions of interest such as MT or MT plus-ends (comets) and the nearby cytosolic areas were measured by the “Intensity Line Profile” tool by drawing a line to extract the intensity distribution profiles. Next, the fluorescence intensity values of the MT region versus the neighboring cytosolic mean intensity were determined to obtain the MT-to-cytosol ratio (*F*_MT_/*F*_cytosol_ or *F*_comet_/*F*_cytosol_). Six to eight various regions per cell were typically chosen to obtain the averaged ratios. Images acquired by the DeltaVision imaging workstation were saved in the tiff format and further processed using ImageJ (NIH) with the MTrackJ plugin. The MT-to-cytosol or comet-to-cytosol analysis was calculated with the command [analyze] – [plot profile]. The collected data were further analyzed or plotted with GraphPad Prism. The apparent half-life time of the fluorescent signal was calculated by using a single-component exponential decay function. The IC_50_ value for the p53-MDM2 inhibitor was determined by using the equation *Y* = bottom + (top – bottom)/(1 + 10^∧^(*X* – log IC_50_)).

### Cell Cycle and Viability Analysis

To evaluate the effects of overexpressing OligoMT on cell cycles, HeLa cells were plated on four 35 mm glass-bottom dishes and transfected with mCherry-tagged OligoMT or mCherry alone (as a negative control). 24 h later, the harvested HeLa cells were washed with PBS and fixed in ice-cold 70% ethanol at 4 °C for 30 min. After being washed with PBS, the cells were treated with 1 mg/mL DAPI (Sigma D9542) and then analyzed by flow cytometry. The histograms of the cell distribution in different cell cycle stages were acquired using a BD LSRII flow cytometer (BD Biosciences). The cell cycle distribution and determination of the fraction of cells in the G0/G1, S, and G2/M phases were analyzed by using FlowJo software. Each sample was assayed in triplicate. Cell viability was assessed using the standard trypan blue staining assay as described previously.^33^

### Statistical Analysis

Quantitative data are presented as the mean and SEM unless otherwise noted. Sample sizes (*n*) were listed for each experiment. Two-tailed Student’s *t* test was used to analyze significant differences between group means. For all statistics, ns, *P* ≥ 0.05; *P < 0.05; **P < 0.01; and ***P < 0.001.

## Data Availability

The data supporting the findings of this study are available from the corresponding author upon request.

## Supporting Information Available

Confocal images and quantifications of OligoMT and/or OligoTIP under different applications (PDF)
3D reconstruction of a HeLa cell transfected with mCherry-OligoMT and high-resolution imaging revealing the microtubule (MT) cytoskeleton clearly marked by mCherry-OligoMT (MP4)
Monitoring mitosis in HeLa cells transfected with H2B-GFP only or cotransfected with mCh-OligoMT (indicator of the MT cytoskeleton) and H2B-GFP (marker of chromatin) with no significant perturbation to the host cell division noted following the coexpression (MP4)
Time-lapse confocal imaging of HeLa cells co-transfected with mCherry-OligoMT and EB1-GFP and EB1-GFP tracking the microtubule (MT) plus-ends and exhibiting movement along MTs, which are distinctly labeled by mCherry-OligoMT (MP4)
Time-lapse confocal imaging of HeLa cells transfected with mCherry-OligoTIP and GFP-OligoMT, OligoTIP tracking the microtubule (MT) plus-ends along MTs, which are clearly labeled by GFP-OligoMT, and co-transfection of mCherry-OligoTIP and GFP-OligoMT enabling real-time visualization of the MT cytoskeleton and MT plus-end dynamics (MP4)
Time-lapse confocal imaging for simultaneous monitoring of microtubule plus-end dynamics in HeLa cells co-transfected with mCherry-OligoTIP and EB1-GFP and mCherry-OligoTIP tracking microtubule (MT) plus-ends and exhibiting constant movement along +TIPs marked by EB1-GFP (MP4)
Screening p53-MDM2 inhibitors with an OligoMT-based assay and confocal images of HeLa cells expressing MDM2-GFP-OligoMT and mCherry-p53 and mCh-p53 interacting with MT-bound MDM2-GFP-OligoMT in the absence of nutlin-3 but translocating from MT toward the cytosol following the addition of 5 μM nutlin-3, an inhibitor that could disrupt the p53-MDM2 association (MP4)
