# Supplementary material for: Genetically Encoded Microtubule Binders for Single-Cell Interrogation of Cytoskeleton Dynamics and Protein Activity
Source: ACS Sens. 2024 Aug 15;9(9):4758–66. doi: 10.1021/acssensors.4c01167 (PMC11443526; doi:10.1021/acssensors.4c01167)
Supplement: Supplementary file 1 — se4c01167_si_001.pdf [file se4c01167_si_001.pdf]

Supporting Information

Genetically encoded microtubule binders for single-cell interrogation of cytoskeleton dynamics and protein activity

Joseph Zhou [1,#], Xiaoxuan Liu[1,#], Dekai Zhang[1,*], Guolin Ma[2*]

[1] Institute of Biosciences and Technology, Texas A&M University, Houston, TX 77030, USA

[2] ORBIT Platform, The University of Texas MD Anderson Cancer Center, Houston 77054, TX, USA

# These authors contributed equally to the work

* Corresponding authors: Dekai Zhang, E-mail: dekaizhang@tamu.edu; Guolin Ma, E-mail: gma@mdanderson.org

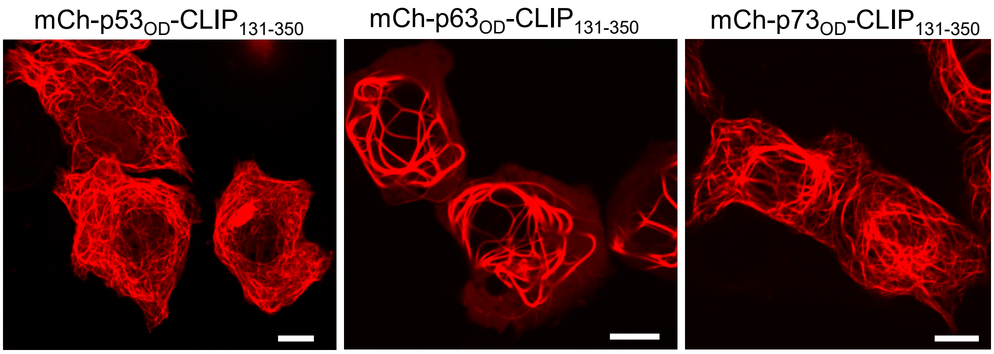

Figure S1 | Characterization of the oligomerization domains from the p53 family protein when fused to the MT binding domain. The confocal images depict the localization of mCherry (mCh)-tagged oligomerization domains (OD), derived from the p53 family (p53, p63, and p73), when fused to the N-terminal microtubule (MT) binding region (aa 131-350) of CLIP170. Scale bar, 10 µm.

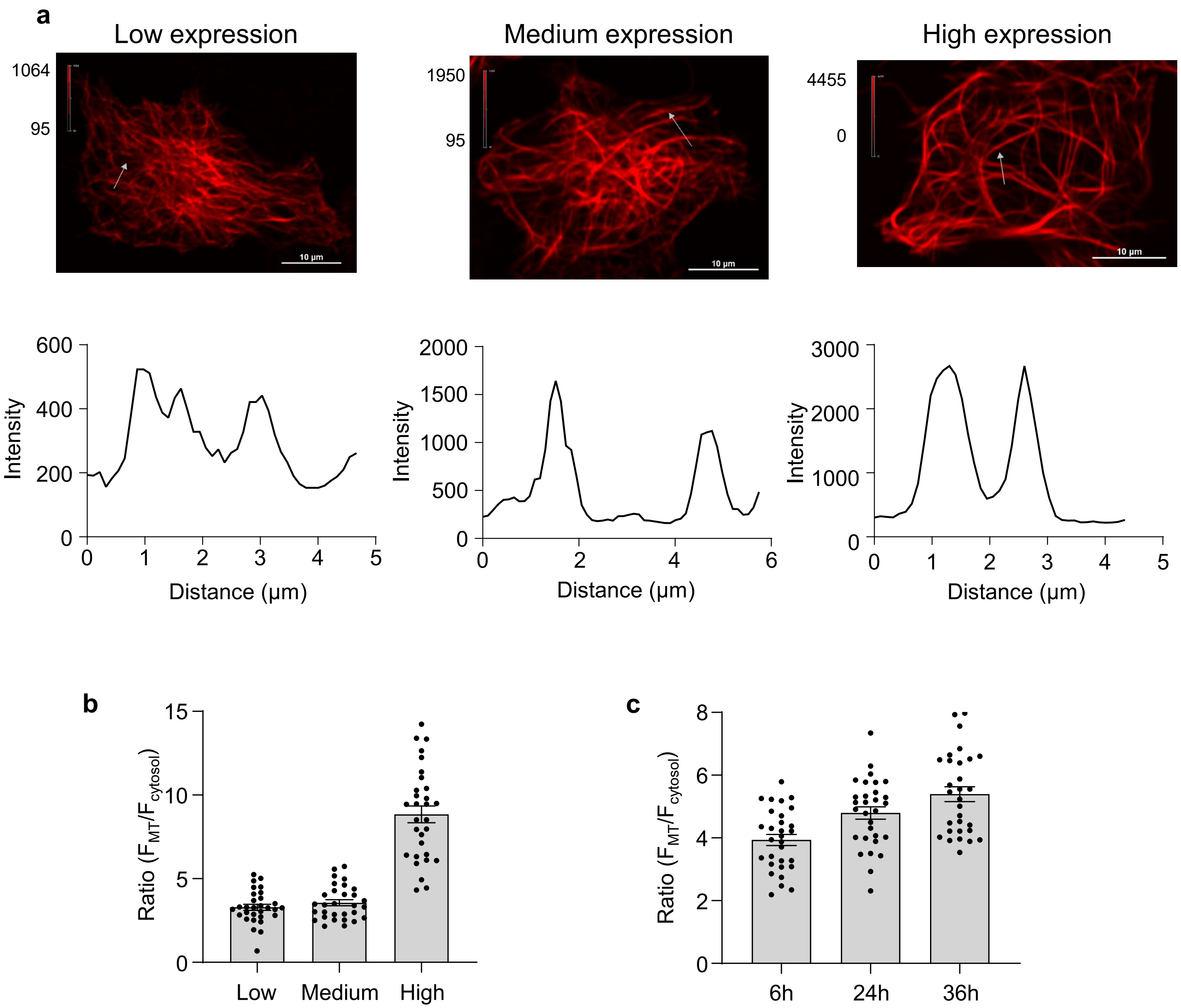

Figure S2 | Statistics of MT-over-cytosol fluorescence ratio ($F_{MT}/F_{cytosol}$) of OligoMT at varying expression levels.

(a) Representative confocal images of HeLa cells expressing OligoMT at low (50 ng plasmid), medium (100 ng) and high (400 ng) levels. The fluorescence intensity range is indicated in the top left corner of each image. The graphs below the images show the fluorescence intensity plot along the white arrows indicated in the corresponding images. Scale bar, 10 μm.

(b) Quantification of the fluorescent intensity ratio ($F_{MT}/F_{cytosol}$) of HeLa cells transfected with the indicated amounts of mCh-OligoMT plasmids. n=30 cells; error bars denote SEM.

(c) Quantification of the fluorescent intensity ratio ($F_{MT}/F_{cytosol}$) of HeLa cells expressing mCh-OligoMT (100 ng plasmid) at the indicated time points after transfection. n=30 cells; error bars denote SEM.

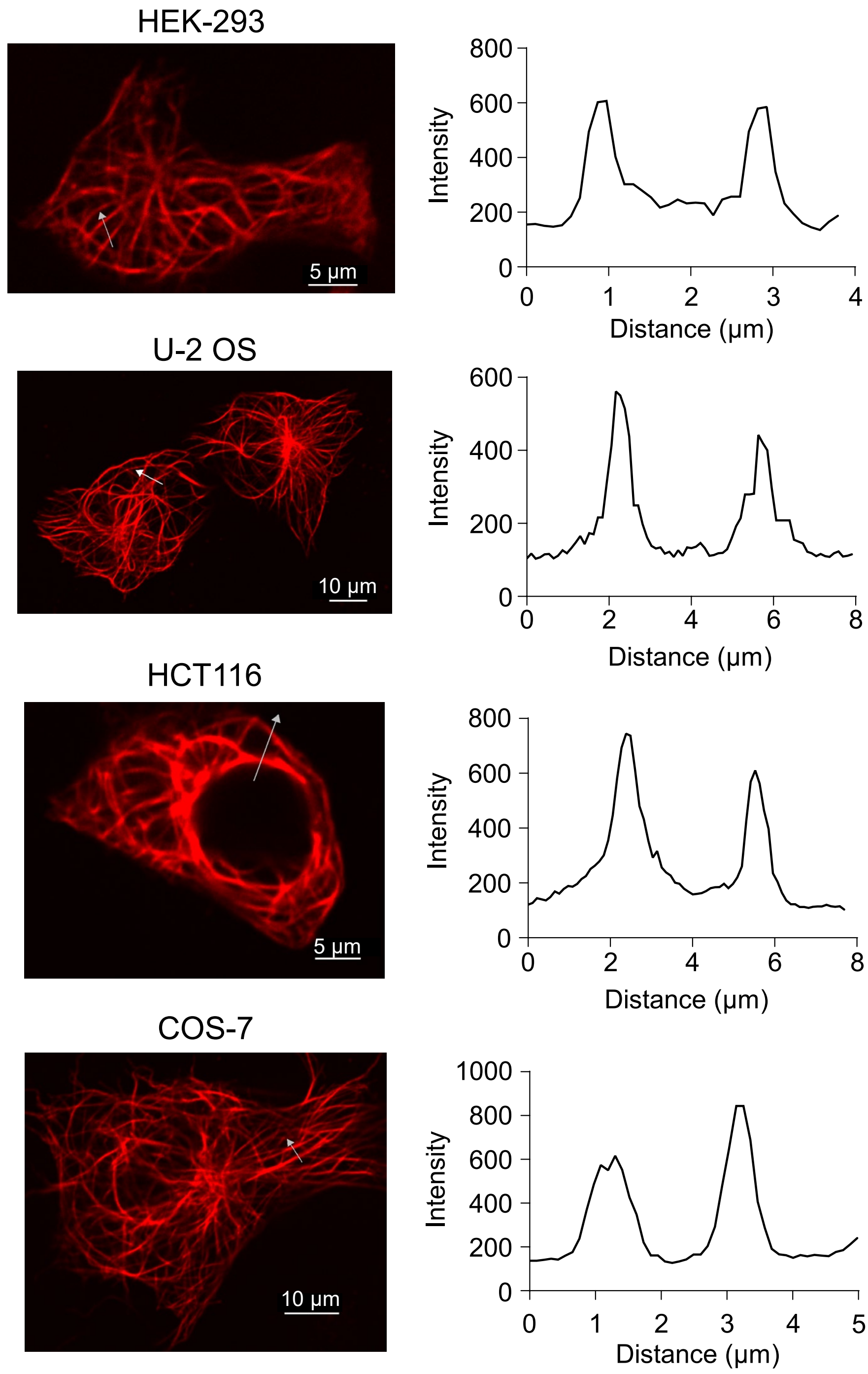

Figure S3 | Confocal images of the indicated cell lines expressing mCh-OligoMT. 100 ng mCh-tagged OligoMT was transfected into HEK-293 (human embryonic kidney cells), U-2 OS (human osteosarcoma cells), HCT116 (human colorectal carcinoma cells), and COS-7 (CV-1 African green monkey kidney fibroblast cells). Images were taken 24 h after transfection. Graphs on the right show the fluorescence intensity plot across the indicated arrows.

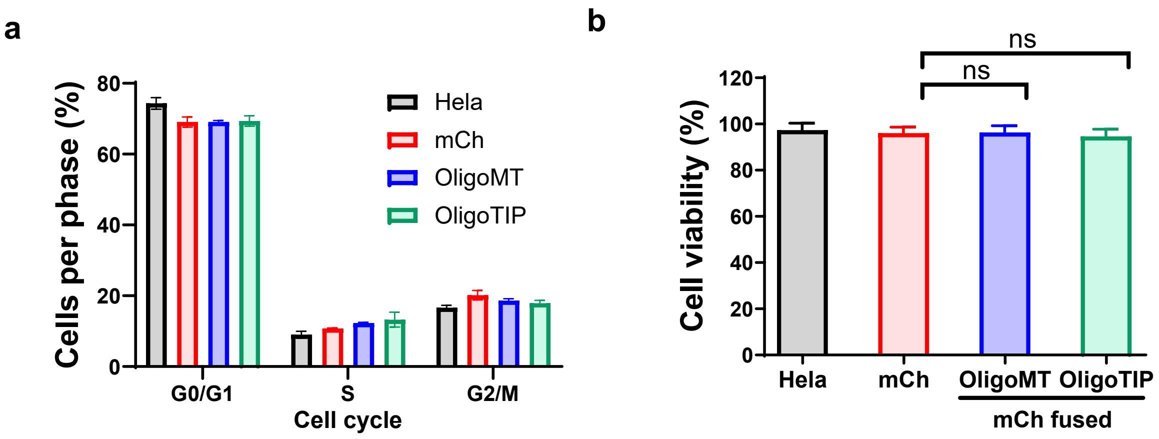

Figure S4 | Cell cycle analysis and viability quantification in cells transfected with OligoMT or OligoTIP.

(a) Cell cycle analysis was performed on HeLa cells transfected with mCh (control), mCh-OligoMT, and mCh-OligoTIP constructs.

(b) Quantification of cell viability was conducted using trypan blue staining 24 hours post-transfection with the indicated constructs. Data are presented as mean ± standard error of the mean (sem) from three independent experiments.

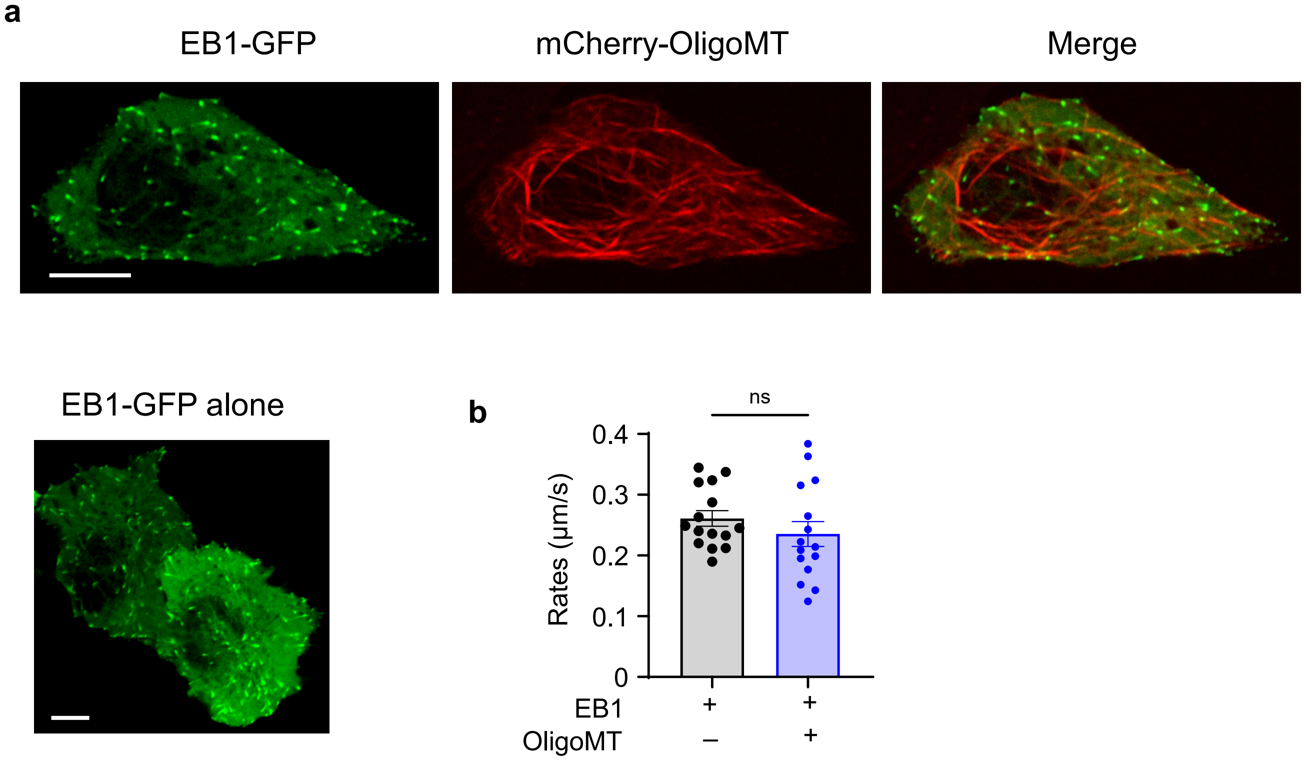

Figure S5 | OligoMT expression does not perturb microtubule plus ends dynamics.

(a) Confocal images depicting EB1-GFP, a highly sensitive marker of dynamic microtubule plus-ends, in the presence (top) and absence (bottom) of coexpressed mCh-OligoMT in HeLa cells.

(b) Quantification of the comet velocity of EB1 with and without coexpression of OligoMT in HeLa cells. n = 15 cells from three independent experiments (mean ± sem; ns, not significant; Paired Student's t-test).

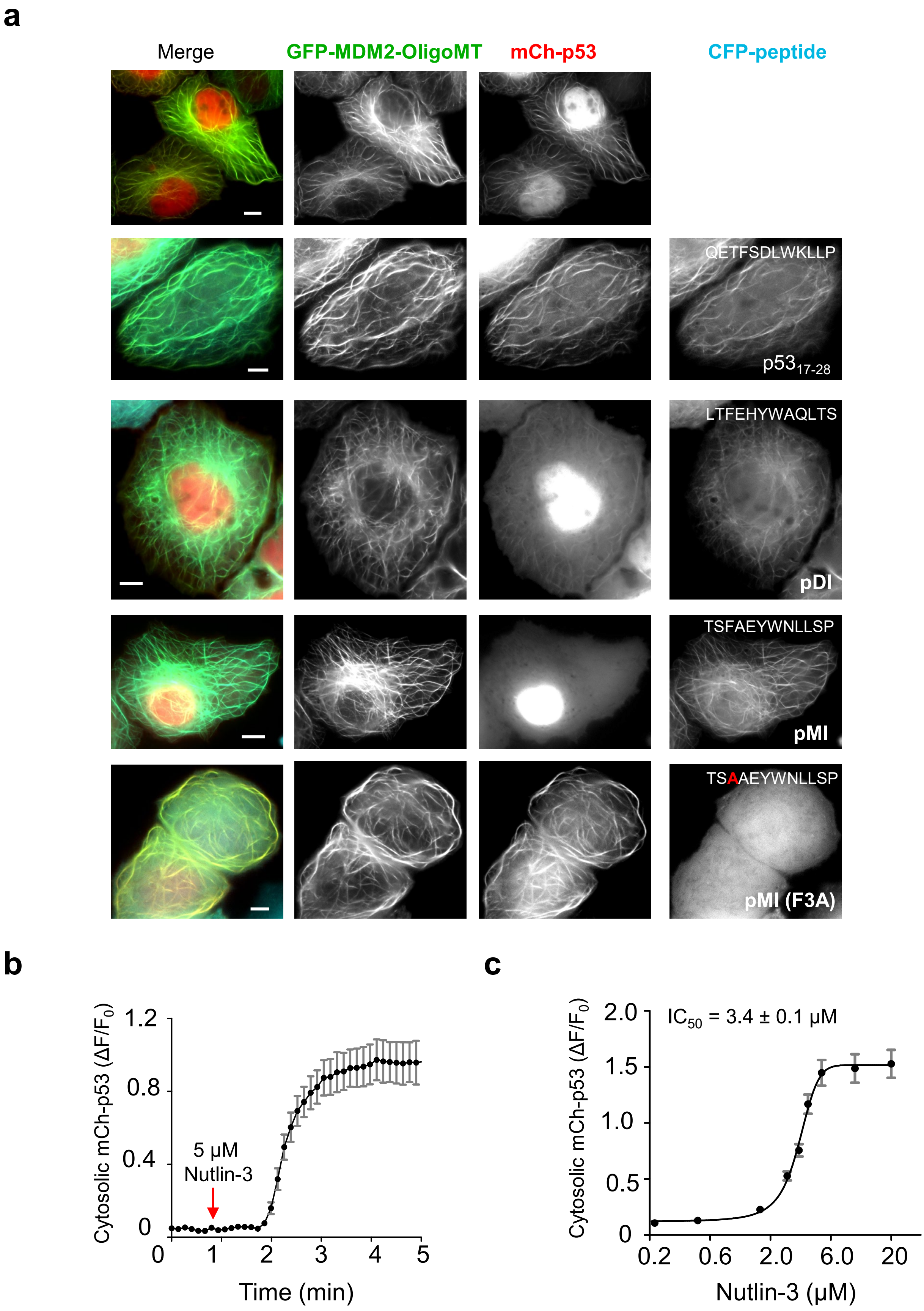
a
Merge
GFP-MDM2-OligoMT
mCh-p53
CFP-peptide
QETFSDLWKLLP
p53 17-28
LTFEHYWAQLTS
pDI
TSFAEYWNLLSP
pMI
TSAAEYWNLLSP
pMI (F3A)
b
Cytosolic mCh-p53 (ΔF/F0)
1.2
0.8
0.4
0
5 μM
Nutlin-3
0
1
2
3
4
5
Time (min)
c
Cytosolic mCh-p53 (ΔF/F0)
IC50 = 3.4 ± 0.1 μM
2.0
1.5
1.0
0.5
0
0.2
0.6
2.0
6.0
20
Nutlin-3 (μM)

Figure S6 | An OligoMT-based assay used to characterize p53-MDM2 inhibitors in living cells.

(**a**) Images of HeLa cells co-expressing MDM2-GFP-OligoMT, mCherry-p53 and CFP tagged inhibitor peptides ($p53_{17\text{-}28}$, pDI, pMI and the pMI-F3A mutant). The cytosolic fraction of mCh-p53 colocalized tightly with MT-bound MDM2-GFP-OligoMT in the absence of the inhibitory peptides. By contrast, in the presence of inhibitor peptides (CFP-pDI or CFP-pMI; bound to MT due to stronger interactions with MDM2), mCh-p53 was displaced from MT and exhibited more even distribution in the cytosol. CFP-pMI-F3A, which displayed a smooth distribution in the cytosol due to disruption of its interaction with p53, was used as a negative control in the assay. Scale bar, 5 μm.

(**b**) Time course showing the release of mCh-p53 from MT toward the cytosol upon addition of a potent MDM2 inhibitor, nutlin-3 (5 μM), within HeLa cells coexpressing mCh-p53 and MDM2-GFP-OligoMT. n = 10 cells from three independent experiments (mean ± sem).

(**c**) A dose-response curve showing nutlin-3-mediated inhibition on p53-MDM2 interaction by using the cytosolic mCh-p53 intensity as a readout. The cytosolic mCherry signals were plotted as a function of titrated nutlin-3 concentrations. n = 31 cells from three independent experiments (mean ± sem).

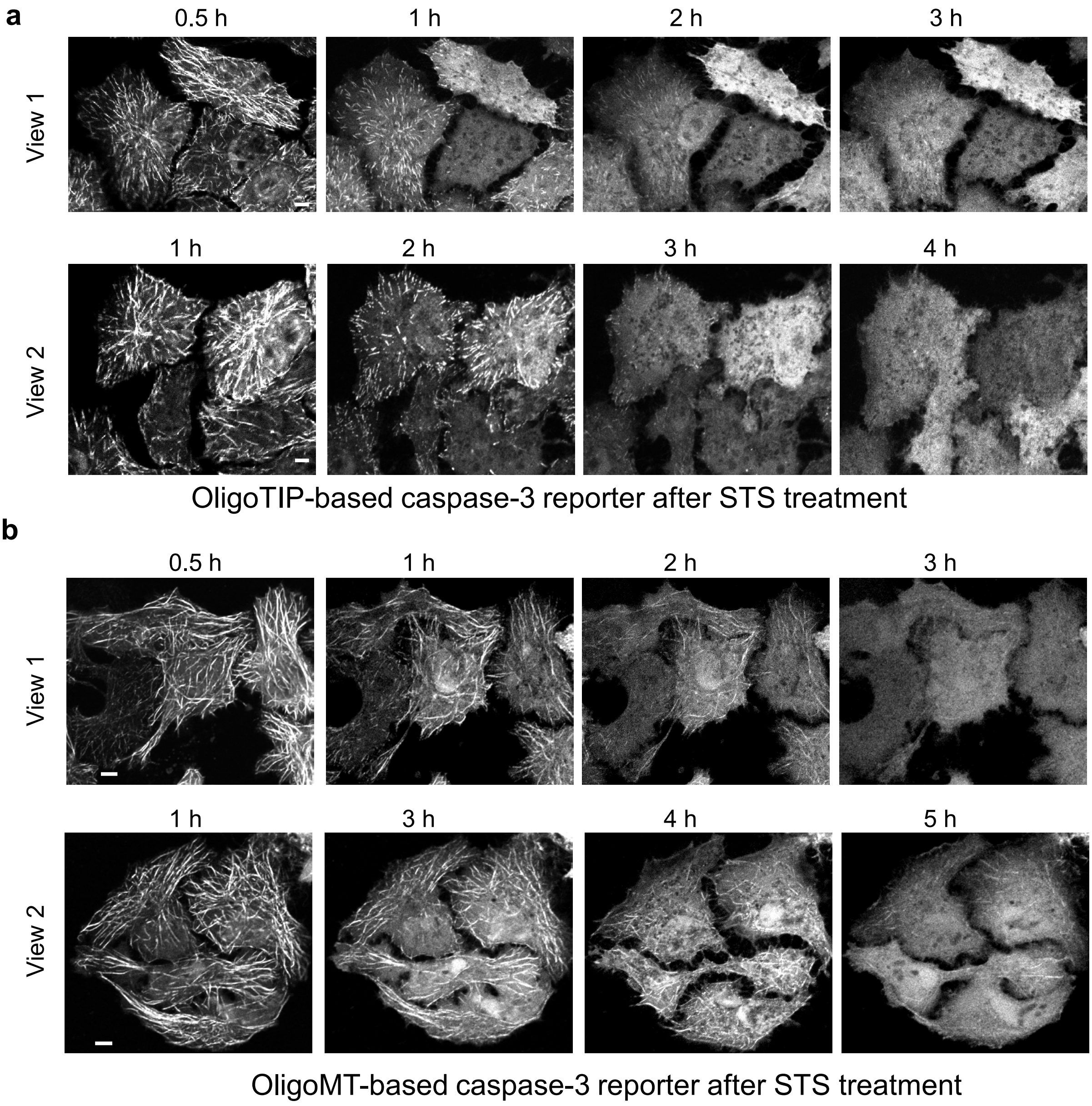

Figure S7 | OligoTIP- and OligoMT-based biosensors to monitor caspase 3 activity in HeLa cells at real time. Shown were two representative views of HeLa cells expressing GFP-DEVD-OligoTIP (**a**) or GFP-DEVD-OligoMT (**b**) at the indicated time points after incubation with 1 μM staurosporine (STS) as an apoptosis inducer. Activated caspase 3 cleaved DEVD to release GFP from MT-bound OligoTIP, thus causing a more even distribution of GFP signals across the cytosol. Scale bar, 5 μm.

Supplementary Videos

Supplementary Video 1. 3D reconstruction of a HeLa cell transfected with mCherry-OligoMT. High-resolution imaging reveals the microtubule (MT) cytoskeleton clearly marked by mCherry-OligoMT.

Supplementary Video 2. Monitoring mitosis in HeLa cells transfected with H2B-GFP only (bottom panel) or co-transfected with mCh-oligoMT (indicator of MT cytoskeleton) and H2B-GFP (marker of chromatin; top panel). No significant perturbation to the host cell division was noted following the co-expression.

Supplementary Video 3. Time-lapse confocal imaging of HeLa Cells co-transfected with mCherry-OligoMT and EB1-GFP. EB1-GFP tracks the microtubule (MT) plus ends and exhibits movement along MTs, which are distinctly labeled by mCherry-OligoMT.

Supplementary Video 4. Time-lapse confocal imaging of HeLa cells transfected with mCherry-OligoTIP and GFP-OligoMT. OligoTIP tracks the microtubule (MT) plus ends along MTs, which are clearly labeled by GFP-OligoMT. The co-transfection of mCherry-OligoTIP and GFP-OligoMT enables real-time visualization of MT cytoskeleton and MT plus end dynamics.

Supplementary Video 5. Time-lapse confocal imaging for simultaneous monitoring of microtubule plus end dynamics in HeLa cells co-transfected with mCherry-OligoTIP and EB1-GFP. mCherry-OligoTIP tracks microtubule (MT) plus ends and exhibits constant movement along +TIPs marked by EB1-GFP.

Supplementary Video 6. Screening p53-MDM2 inhibitors with an OligoMT-based assay. Shown were the confocal images of HeLa cells expressing MDM2-GFP-OligoMT (middle) and mCherry-p53 (right). mCh-p53 interacted with MT-bound MDM2-GFP-OligoMT in the absence of nutlin-3, but translocated from MT toward the cytosol following the addition of 5 μM nutlin-3, an inhibitor that could disrupt the p53-MDM2 association.
